# Intermittent Fasting‐Induced Orm2 Promotes Adipose Browning via the GP130/IL23R‐p38 Cascade

**DOI:** 10.1002/advs.202407789

**Published:** 2024-09-09

**Authors:** Xuejuan Zhu, Xinran Wang, Jingang Wang, Lei Du, Zhen‐Ning Zhang, Donglei Zhou, Junfeng Han, Bing Luan

**Affiliations:** ^1^ Department of Endocrinology Tongji Hospital Affiliated to Tongji University School of Medicine Tongji University Shanghai 200092 China; ^2^ Department of Breast and Thyroid Surgery Shanghai Tenth People's Hospital School of Medicine Tongji University Shanghai 200072 China; ^3^ Translational Medical Center for Stem Cell Therapy & Institute for Regenerative Medicine Shanghai East Hospital School of Life Sciences and Technology Tongji University Shanghai 200092 China; ^4^ Department of Gastric Surgery Fudan University Shanghai Cancer Center Shanghai 200032 China; ^5^ Department of Oncology Shanghai Medical College Fudan University Shanghai 200032 China

**Keywords:** adipose tissue browning, GP130/IL23R, intermittent fasting, Obesity, Orm2

## Abstract

Intermittent fasting (IF) plays a critical role in mitigating obesity, yet the precise biological mechanisms require further elucidation. Here Orosomucoid 2 (Orm2) is identified as an IF‐induced hepatokine that stimulates adipose browning. IF induced Orm2 expression and secretion from the liver through peroxisome proliferator‐activated receptor alpha (PPAR*α)*. In adipose tissue, Orm2 bound to glycoprotein 130/interleukin 23 receptor (GP130/IL23R) and promoted adipose browning through the activation of p38 mitogen‐activated protein kinases (p38‐MAPK). In obese mice, Orm2 led to a significant induction of adipose tissue browning and subsequent weight loss, an effect that is not replicated by a mutant variant of Orm2 deficient in GP130/IL23R binding capability. Crucially, genetic association studies in humans identified an obesity‐associated Orm2 variant (D178E), which shows decreased GP130/IL23R binding and impaired browning capacity in mice. Overall, the research identifies Orm2 as a promising therapeutic target for obesity, mediating adipose browning through the GP130/IL23R‐p38 signalling pathway.

## Graphical abstract

During intermittent fasting, peroxisome proliferator‐activated receptor alpha (PPARα) in the liver binds to the peroxisome proliferator response element (PPRE), leading to the upregulation of Orm2. Orm2 then interacts with the glycoprotein 130/interleukin 23 receptor (GP130/IL23R) complex on adipocytes, initiating the activation of p38 mitogen‐activated protein kinase (p38 MAP kinase). This sequence boosts the activity of uncoupling protein 1 (UCP1), thereby facilitating thermogenesis in adipose tissue.

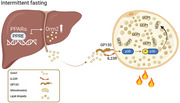

## 1 Introduction

The escalating incidence of obesity and associated metabolic pathologies, such as type 2 diabetes and atherosclerosis, can primarily be ascribed to a disequilibrium between caloric intake and energy expenditure. Accumulated evidence suggests that non‐shivering thermogenesis restores energy balance by promoting lipolysis and dissipating energy as heat rather than through ATP synthesis, in both Uncoupling protein 1 (Ucp1)‐dependent and Ucp1‐independent manners.^[^ ^1^, ^2^, ^3^, ^4^, ^5^ ^]^ Therefore, brown and beige adipose tissues, collectively termed thermogenic adipose tissues, are increasingly recognized as distinctive targets for the alleviation of metabolic diseases associated with obesity.

Intermittent fasting (IF) is recognized for mitigating metabolic disorders and yielding a range of health benefits.^[^ ^6^, ^7^ ^]^ The beneficial effects of IF in combating obesity, cardiovascular diseases, aging, cancer and neurodegenerative diseases are well documented in both clinical settings and animal models.^[^ ^6^, ^8^, ^9^, ^10^ ^]^ IF has been shown to confer benefits to energy homeostasis by augmenting energy expenditure in mammalian models. In a previous study, Kim et al., demonstrated that IF induces an upregulation of vascular endothelial growth factor in white adipose tissue (WAT), which plays a critical role in macrophage polarization and the browning process of WAT.^[^ ^11^ ^]^ Additionally, IF has been implicated in promoting WAT browning through the fermentation products of gut microbiota‐specifically acetate and lactate.^[^ ^12^ ^]^ Although pregnancy zone protein, a hepatokine secreted from the liver, stimulates fuel uptake in brown adipose tissue (BAT) and supports thermogenesis during refeeding, the intricate communication between the liver and thermogenic adipose tissue during IF is yet to be fully elucidated.^[^ ^13^ ^]^

The Orosomucoid (Orm) family, consisting of acute‐phase secretory glycoproteins, plays a pivotal role in modulating inflammation, immune responses and energy metabolism.^[^ ^14^, ^15^, ^16^, ^17^ ^]^ In human serum, there are two ORM isoforms, ORM1 and ORM2, whereas in mice, there are three variants: Orm1, Orm2, and Orm3.^[^ ^18^ ^]^ Orm2, predominantly synthesized in the liver, is upregulated in response to various stimuli, including acute infections, inflammation, neoplastic conditions, sepsis, and tissue damage.^[^ ^19^ ^]^ Recent studies have highlighted Orm2's critical involvement in hepatic lipid metabolism and obesity. It has been shown that Orm2 can activate AMP‐activated protein kinase (AMPK) signaling pathways and suppress de novo lipogenesis, thereby mitigating liver steatosis and associated lipid abnormalities.^[^ ^20^ ^]^ Moreover, Orm2 can decrease fatty acid absorption by downregulating the Erk1/2‐PPARγ‐CD36 axis, contributing to the improvement of hepatic steatosis.^[^ ^21^ ^]^ A deficiency in Orm2 exacerbates obesity induced by a high‐fat diet through mechanisms involving alterations in gut microbiota and an increase in intestinal inflammation.^[^ ^22^ ^]^ These findings collectively suggest a significant role for Orm2 in metabolic homeostasis. Nevertheless, the potential involvement of Orm2 in thermogenic regulation as a means to counteract obesity has not yet been fully established.

In this study, we have discovered that IF stimulates the expression and secretion of Orm2 from the liver via activation of the PPAR*α*. This hepatically derived Orm2 then circulates to adipose tissue, where it interacts with the glycoprotein 130/interleukin 23 receptor (GP130/IL23R) complex. Such binding leads to the enhancement of adipose tissue's thermogenic capacity through the activation of the p38 mitogen‐activated protein kinases (p38 MAPK) signaling pathway. Notably, in the context of obesity, Orm2, but not a GP130/IL23R‐binding deficient mutant of Orm2, is capable of inducing thermogenesis. Therefore, we delineate an Orm2‐GP130/IL23R‐p38 signaling cascade as a novel molecular link between hepatic function and adipose tissue thermogenesis in response to IF. These findings suggest that Orm2 holds promise as a novel therapeutic target for the treatment of obesity.

## 2 Results

### 2.1 IF Induces Orm2 Secretion Through PPAR*α* in Liver

**Figure 1:**
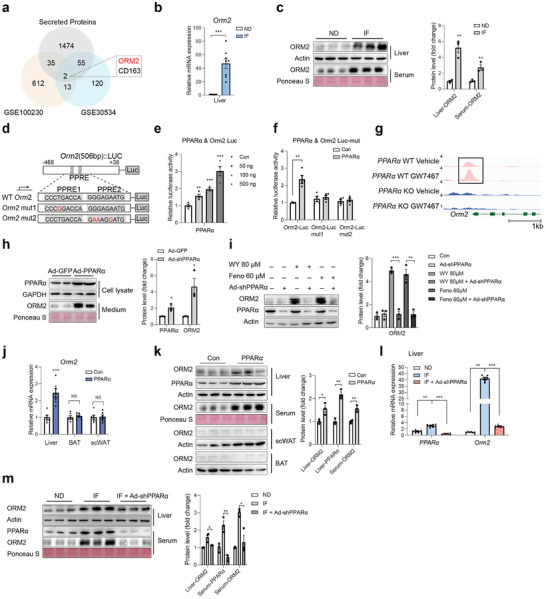
IF induces Orm2 secretion through PPAR*α* in liver. a) Diagram of genes annotated as secreted proteins that significantly changed in liver of long‐term and short‐term IF‐treated mice. b) Orm2 mRNA levels in liver, BAT and scWAT from normal diet (ND) or Intermittent fasting (IF) mice (n = 8). c) Orm2 protein levels in liver, serum, scWAT, and BAT from normal diet (ND) or Intermittent fasting (IF) mice. d) Scheme illustrating the luciferase reporter plasmid containing Orm2 promoter region with predicted PPAR*α* binding site and mutated sites (mut1 and mut2). e,f) Effect of PPAR*α* on Orm2 (e) or Orm2‐mut (f) luciferase reporter activity (n = 3). g) Alignment of PPAR*α* Chip‐seq peaks at Orm2 gene locus in liver. h) Orm2 protein levels in medium of primary hepatocytes infected with Ad‐GFP and Ad‐PPAR*α*. i) Orm2 protein levels in primary hepatocytes treated with PPAR*α* activator WY14643 (80 µm) or Fenofibrate (60 µm) after infection with Ad‐shPPAR*α* or Ad‐shNC. j) Orm2 mRNA levels in liver, BAT and scWAT in control or PPAR*α* plasmid‐injected mice (n = 6). k) Orm2 protein levels in liver, serum, scWAT, and BAT from control or PPAR*α* plasmid‐injected mice. l) Representative PPAR*α* and Orm2 expression in liver from ND, IF, and IF mice infected with Ad‐shPPAR*α* (n = 6). m) Orm2 protein levels in liver and serum from ND, IF and IF mice infected with Ad‐shPPAR*α*. Significance was calculated by unpaired two‐tailed Student's t test (b, c, e, f, h, i, j, k, m) or two‐way ANOVA (l) *p < 0.05, **p < 0.01, ***p < 0.001 and ****p < 0.0001 were considered to be significant. Error bars represent the mean ± SEM.

The liver is recognized as a key organ that is responsive to IF.^[^ ^7^ ^]^ The potential role of hepatokines secreted by the liver in facilitating adipose tissue browning during IF is not yet fully understood. To explore IF‐regulated hepatokines that may influence this process, we focused on long‐term and short‐term IF‐upregulated transcripts (NCBI GEO: GSE30534 and GSE100230) in the liver. By integrating these datasets with data on secreted proteins in whole mice, we aimed to identify liver‐specific secreted factors (*p* < 0.05, Log_2_FC > 1). From this analysis, Orm2 was identified as a noteworthy candidate (**Figure** **1**a). Orm2 is a secretory factor highly expressed in the liver, and with trace amounts of expression in other tissues including adipose tissue (Figure S1a,b, Supporting Information). We next adopted an IF protocol of every‐other‐day fasting (Figure S1c, Supporting Information), which has been reported to promote adipose browning,^[^ ^23^ ^]^ to confirm this result. Indeed, four‐cycle IF led to increased adipose browning, as indicated by augmented oxygen consumption (VO_2_), a lower respiratory exchange ratio (RER), and elevated energy expenditure (EE) (Figure S1d‐f, Supporting Information). Furthermore, there was an increased expression of thermogenic genes such as Ucp1, Pgc1*α*, Prdm16, and Cox8b, as well as elevated Ucp1 protein levels in subcutaneous white adipose tissue (scWAT) (Figure S1g‐i, Supporting Information). Thermogenic gene expression was also moderately increased in BAT, but to a lesser extent compared to that in scWAT (Figure S1j‐l, Supporting Information). Under these circumstances, body weight, adipose tissue weight, cumulative food intake and activity were not influenced significantly (Figure S1m‐p, Supporting Information). We assessed Orm2 expression in the liver from IF‐treated mice compared with ad libitum (AL)‐treated mice (ND). In line with expectations, both mRNA and protein levels of Orm2 were elevated in the liver following IF. (Figure 1b,c). Correspondingly, serum Orm2 levels were also higher in IF‐treated mice, suggesting enhanced hepatic secretion of Orm2 in response to IF (Figure 1c). Meanwhile, we examined the expression of Orm2 across various tissues in IF mice and found that Orm2 was significantly elevated only in the liver, with no notable changes in other tissues (Figure S1q, Supporting Information). Additionally, we investigated two other genes in the Orm family, Orm1 and Orm3, and discovered that only Orm2 showed significant upregulation in the liver of IF mice (Figure S1r, Supporting Information). Peroxisome proliferator‐activated receptor *α* (PPAR*α*) has been reported to be a key transcriptional factor in response to IF treatment in the liver, and in our study, we also observed increased expression of PPAR*α* in the liver under IF conditions. (Figure S1s, Supporting Information).^[^ ^24^ ^]^ Additionally, we examined known downstream targets of PPAR*α* and found that genes such as Acox1, CD36, and Cpt2 were also upregulated (Figure S1t, Supporting Information). We used the database Genomatix (GSE61817) to predict PPAR*α* binding sites in the Orm2 promoter and identified two putative sequences (Figure 1d). Luciferase reporter assays with vectors containing the wild‐type Orm2 promoter demonstrated increased activity when co‐expressed with PPAR*α*, but this effect was not seen with vectors containing mutations in the PPAR*α* binding sites (Figure 1e,f). Further, Chromatin Immunoprecipitation (ChIP)‐sequencing results revealed binding peaks at these putative sites that were enhanced by the PPAR*α* agonist GW7647 treatment, confirming direct binding of PPAR*α* to the Orm2 promoter (Figure 1g). As a result, overexpression of PPAR*α* in primary hepatocytes using an adenovirus vector (Ad‐PPAR*α*) enhanced Orm2 expression and secretion (Figure 1h; Figure S1u, Supporting Information). Treatment with PPAR*α* activators, WY14643 (80 µm) or Fenofibrate (60 µm), also increased Orm2 expression and secretion, effects that were inhibited by a PPAR*α* knockdown using adenovirus‐mediated shRNA (Ad‐shPPAR*α*) (Figure 1i; Figure S1v, Supporting Information). To confirm this regulatory mechanism in vivo, we administered intravenous injections of a PPAR*α* expression plasmid to C57BL/6J mice (Figure 1j), which resulted in increased Orm2 expression in the liver and serum, but not in BAT or scWAT (Figure 1k). Conversely, intravenous injection of Ad‐shPPAR*α*, which knocked down PPAR*α* expression in the liver, abolished the IF‐induced increases in Orm2 expression and secretion (Figure 1lm). These findings collectively suggest that IF prompts Orm2 secretion in the liver via PPAR*α* activation.

### 2.2 Orm2 Promotes Adipose Browning

**Figure 2:**
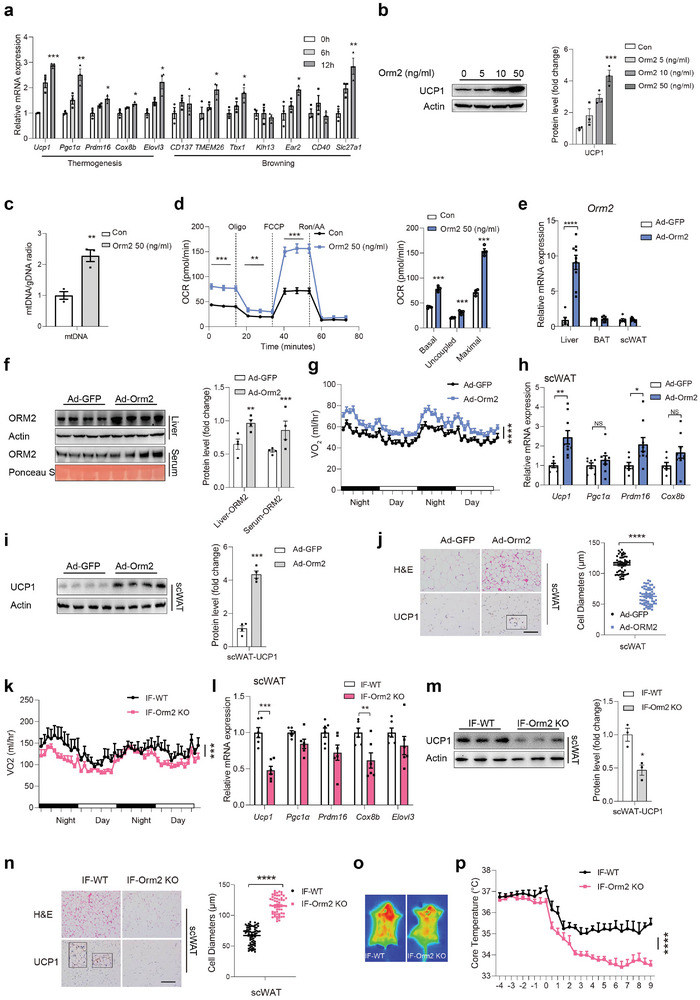
Orm2 promotes adipose browning. a) Representative thermogenic genes and browning‐related genes mRNA levels in immortalized brown adipocytes (BAC) treated with 50 ng mL^−1^ Orm2 (n = 3). b) Ucp1 protein levels in BAC treated with indicated concentrations of Orm2. c) mtDNA Content in SVF cells after Orm2 (50 ng mL^−1^) treatment for 24 h (n = 3). d) Oxygen consumption rate (OCR) in SVF cells was determined by Seahorse after treatment with Orm2 (50 ng mL^−1^) for 24 h (n = 3). e–j) C57BL/6 mice were intravenously injected with Ad‐GFP or Ad‐Orm2 for 1 week. Orm2 mRNA levels in liver, BAT and scWAT (e; n = 6/9), Orm2 protein levels in liver and serum (f; n = 4), Oxygen consumption (VO_2_) (g; n = 6/8), thermogenic genes mRNA levels of scWAT (h; n = 7/9), Ucp1 protein levels of scWAT (i), H&E and Ucp1 staining of scWAT (j; Scale bar,100 µm, n = 3) were detected. k–n) Orm2‐KO mice and WT littermates were subjected to IF for 12 days. Oxygen consumption (VO_2_) (k; n = 6), thermogenic genes mRNA levels of scWAT (l; n = 6), Ucp1 protein levels of scWAT (m), H&E and Ucp1 staining of scWAT (n; Scale bar,100 µm, n = 3) were detected. o,p) Infrared thermography in WT or Orm2‐KO mice that were subjected to IF for 12 days (o; n = 3); Core temperature after exposure to 4 °C in WT and Orm2‐KO mice (p; n = 6). Significance was calculated by unpaired two‐tailed Student's t test (a‐f, h, i, j, l, m, n), two‐way ANOVA (p) or two‐sided ANCOVA (g, k); *p < 0.05, **p < 0.01, ***p < 0.001 and ****p < 0.0001 were considered to be significant. Error bars represent the mean ± SEM.

We next sought to explore whether liver‐secreted Orm2 mediated IF‐induced adipose browning. Hepa1‐6 cells were treated with WY14643 or Fenofibrate, and conditioned medium (CM) was collected and applied to brown/beige adipocyte cell lines (BAC) (Figure S2a, Supporting Information). Medium from WY14643 or Fenofibrate‐treated cells promoted thermogenic gene expression, which was abolished by Orm2 antibody pretreatment (Figure S2b‐d, Supporting Information). Furthermore, CM from Ad‐PPAR*α* or Ad‐Orm2 infected Hepa1‐6 cells also increased thermogenic gene expression in BAC and Orm2 antibody pretreatment abolished this effect (Figure S2e‐g, Supporting Information), indicating that Orm2 secretion from hepatocytes is critical for the regulation of adipose thermogenic gene expression. Indeed, when recombinant Orm2 was applied directly to BAC cells, thermogenic and browning‐related gene expression was promoted in a time‐ and dose‐dependent manner (**Figure** **2**a,b). Additionally, mtDNA content, oxidative phosphorylation genes expression, and OCR data indicated that Orm2 could enhance mitochondrial function in stromal vascular fraction (SVF)‐differentiated primary beige adipocytes (Figure 2c,d; Figure S2h, Supporting Information). FGF21 is well‐documented, liver‐secreted factor that can stimulate adipose thermogenesis.^[^ ^25^, ^26^, ^27^ ^]^ FGF21 is also a PPAR*α* target that increases in liver under IF treatment.^[^ ^25^ ^]^ Indeed, FGF21 antibody also suppressed thermogenic gene expression in BAC treated with CM from WY14643 or Fenofibrate‐treated cells, however, Orm2 antibody treatment augmented this effect on top of FGF21 antibody (Figure S2i‐k, Supporting Information), indicating that FGF21 and Orm2 functioned in parallel to mediate IF‐induced adipose thermogenesis. Additionally, we treated BAC cell lines with recombinant FGF21 and ORM2 proteins or performed local injections into the scWAT of mice. The results showed that Orm2 enhanced the expression of thermogenic genes induced by FGF21, indicating a synergistic interaction between the two proteins (Figure S2l,m, Supporting Information).

To explore the in vivo function of liver‐secreted Orm2, we administered intravenous injections of adenovirus carrying the Ad‐Orm2 to the liver of C57BL/6J mice. This procedure led to increased levels of Orm2 protein in the liver and serum, but not in scWAT or BAT (Figure 2e,f). One week after injection, the mice were subjected to whole‐body indirect calorimetry analysis. Elevated hepatic Orm2 expression was associated with an increase in VO_2_, energy expenditure (EE) and thermogenic gene expression in scWAT, while RER was slightly decreased. (Figure 2g,h; Figure S3a,b, Supporting Information). Additionally, Ucp1 protein levels in scWAT were upregulated, as confirmed by immunoblotting and immunohistochemistry (Figure 2i,j). Adipose tissue mass, cumulative food intake and activity were unaffected (Figure S3c‐e, Supporting Information).

Conversely, we generated Orm2‐KO mice, which exhibited a significant decrease in hepatic Orm2 levels (Figure S3f, Supporting Information). Meanwhile, whole‐body indirect calorimetry analyses revealed downregulated oxygen consumption (VO_2_), energy expenditure (EE), thermogenic gene expression, and Ucp1 protein levels in scWAT from Orm2 KO mice under IF compared to wild‐type littermates, accompanied by a significant reduction in hepatic Orm2 levels, with no change in RER, body weight, food intake, tissue mass, or physical activity (Figure 2k‐n; Figure S3g‐l, Supporting Information). Furthermore, interscapular temperature was decreased in Orm2 KO mice and impaired tolerance to cold (4 °C) was observed, while under thermoneutral conditions, Orm2‐KO mice showed no change (Figure 2o,p; Figure S3m,n, Supporting Information). We further discovered that under cold conditions (4 °C), Orm2 levels gradually increased in liver (Figure S3o, Supporting Information). Additionally, a rise in serum lipolytic products from adipose tissue, including NEFA, glycerol, and ketone bodies at 4 °C, was noted in WT but decreased in Orm2‐KO mice (Figure S3p‐r, Supporting Information), indicating that hepatic ORM2 also promotes cold‐induced thermogenesis. Meanwhile, the absence of Orm2 in primary beige adipocytes did not affect *β*_3_AR agonist CL316243‐induced Ucp1 expression, ruling out the possibility that Orm2 intrinsic to adipocytes impacts thermogenic capacity independently (Figure S3s, Supporting Information). These data indicated that liver‐secreted Orm2 promotes adipose browning and thermogenesis.

### 2.3 Orm2 Functions Through IL23 Receptor‐GP130‐p38

**Figure 3:**
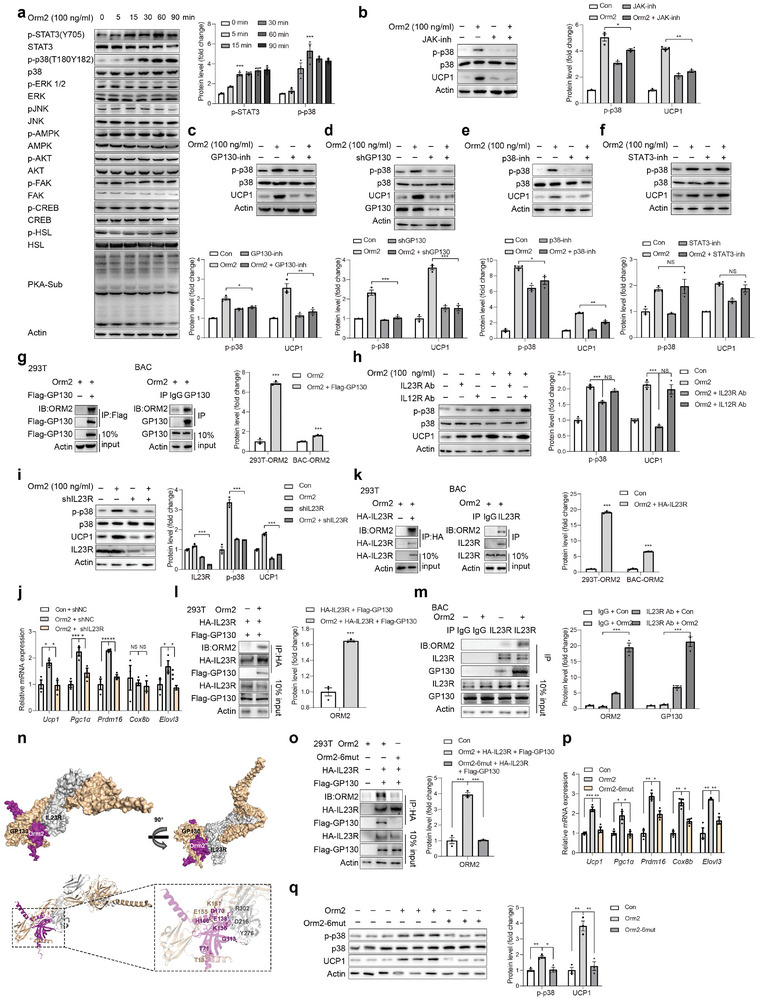
Orm2 functions through IL23 receptor‐GP130‐p38. a) Representative immunoblots of STAT3, p38, ERK, JNK, AMPK, AKT, FAK, CREB, HSL and PKA‐sub signalling pathways in BAC treated with Orm2 (100 ng mL^−1^). b–d) Representative immunoblots of p38 phosphorylation and Ucp1 protein levels in BAC incubated with Orm2 (100 ng mL^−1^) and JAK inhibitor GLPG0634 10 nm (b), GP130 inhibitor SC144 1 µm (c) or infected with Lentivirus‐shGP130 (d). e,f) Representative immunoblots of p38 phosphorylation and Ucp1 protein levels in BAC treated with p38 inhibitor SB202190 (2 µm) (e) and STAT3 inhibitor C188‐9 (1 µm) (f). g) Co‐immunoprecipitation analysis showing the interaction between Orm2 and GP130 in HEK 293T cells and BAC. h) Representative immunoblots of p38 phosphorylation and Ucp1 protein levels in BAC incubated with IgG, IL23R antibody, or IL12R antibody and treated with or without Orm2 (100 ng mL^−1^). i) Representative immunoblots of p38 phosphorylation and Ucp1 protein levels in BAC infected with AAV‐shIL23R and treated with or without Orm2 (100 ng mL^−1^). j) Representative thermogenic genes mRNA levels in BAC infected with AAV‐IL23R and treated with or without Orm2 (100 ng mL^−1^) (n = 3). k) Co‐immunoprecipitation analysis shows the binding of Orm2 with IL23R in HEK 293T cells and BAC. l,m) Co‐immunoprecipitation analysis showing the interaction between IL23R and GP130 with or without Orm2 (100 ng mL^−1^) treatment in HEK 293T cells (l) and BAC (m). n) Space‐filling structural model of the Orm2/GP130/IL23R complex. Orm2 is in magenta, GP130 is in beige, IL23R is in gray. The predicted structures of Orm2, IL23R and GP130 were generated by Alphafold. o) Co‐immunoprecipitation analysis showing the interaction between Orm2 or Orm2‐6mut with transfected IL23R and GP130 in HEK 293T cells. p) Representative immunoblots of p38 phosphorylation and Ucp1 protein levels in BAC incubated with Orm2 or Orm2‐6mut recombinant protein (100 ng mL^−1^). q) Representative thermogenic genes mRNA levels in BAC incubated with Orm2 or Orm2‐6mut recombinant protein (100 ng mL^−1^) (n = 3). Significance was calculated by unpaired two‐tailed Student's *t* test (a‐m, o‐q); *p < 0.05, **p < 0.01 and ***p < 0.001 were considered to be significant. Error bars represent the mean ± SEM.

We next attempted to identify the Orm2 receptor on adipocytes that mediates its effect. Given that previous reports have identified the Leptin receptor (LepR) and C‐C motif chemokine receptor 5 (CCR5), as well as Toll‐like receptor 4 (TLR4) as receptors for Orm1, another Orm family member, we initially investigated whether Orm2 could function through these receptors.^[^ ^17^, ^28^, ^29^ ^]^ Unfortunately, neutralizing these receptors with antibodies or inhibitors did not affect Orm2‐stimulated Ucp1 expression (Figure S4a, Supporting Information). Subsequently, we assessed the impact of Orm2 on various signalling pathways in BAC cells, including JAK2‐STAT3, MAPKs, AKT, AMPK, and cAMP. Intriguingly, we observed that Orm2 treatment stimulated the phosphorylation of STAT3 and p38, but not other kinases (**Figure** **3**a). It is reported that GP130 cytokines including IL‐6, leukemia inhibitory factor (LIF), oncostatin M (OSM), ciliary neurotrophic factor (CNTF), cardiotrophin‐1 (CT‐1), interleukin‐11 (IL‐11), and interleukin‐27 (IL‐27) stimulate STAT3 and p38 phosphorylation at the same time through JAK, which promoted us to investigate whether Orm2 functioned through this system.^[^ ^30^, ^31^, ^32^, ^33^, ^34^, ^35^, ^36^ ^]^ Indeed, pretreatment of BAC cells with JAK inhibitor GLPG0634, GP130 inhibitor SC144 or knockdown of GP130 expression through shRNA transfection abolished Orm2‐induced p38 phosphorylation and Ucp1 expression (Figure 3b‐d; Figure S4b‐d, Supporting Information). Meanwhile, p38 inhibitor but not STAT3 inhibitor abolished Orm2‐stimulated Ucp1 expression (Figure 3e,f), suggesting that Orm2 functioned through GP130‐JAK‐p38 cascade. Co‐immunoprecipitation experiment confirmed the interaction between Orm2 and GP130 both in HEK293T cells transfected with Flag‐GP130 or in BAC cells with endogenous GP130 (Figure 3g). Each GP130 cytokine typically binds to its unique *α*‐subunit receptor, followed by the dimerization of *α* receptor with GP130 to activate downstream signalling and 8 *α* receptors including IL6R*α*, IL11R*α*, CNTFR, LIFR, OSMR, IL27R, IL12R and IL23R have been identified.^[^ ^37^ ^]^ To pinpoint the specific *α* receptor that mediates Orm2's function, we blocked each *α* receptor in BAC cells using corresponding antibodies. Our findings demonstrated that blocking IL23R with an antibody or shRNA (shIL23R) inhibited Orm2's ability to induce phosphorylation of p38 and Ucp1 expression (Figure 3h‐j; Figure S4e, Supporting Information), thereby implicating IL23R as the *α* receptor for Orm2. Co‐immunoprecipitation confirmed the interaction between IL23R and Orm2 in both HEK293T cells and BAC cells (Figure 3k). Additionally, Orm2 facilitated the association between IL23R and GP130 (Figure 3l,m), indicating receptor activation. We employed molecular docking to delineate the interacting regions of Orm2 with IL23R and GP130, revealing that residues T71, H166, D170, K136, G113, and E138 in Orm2 are likely involved in these interactions (Figure 3n). Indeed, Orm2‐6mut (T71A, H166A, D170A, K136A, G113A and E138A) abrogated its interaction with both IL23R and GP130 (Figure 3o). Consistently, wild‐type Orm2 but not Orm2‐6mut, enhanced p38 phosphorylation, Ucp1 expression, and thermogenic gene expression in BAC cells (Figure 3p,q).

### 2.4 Orm2 Promotes Adipose Browning Through IL23R

**Figure 4:**
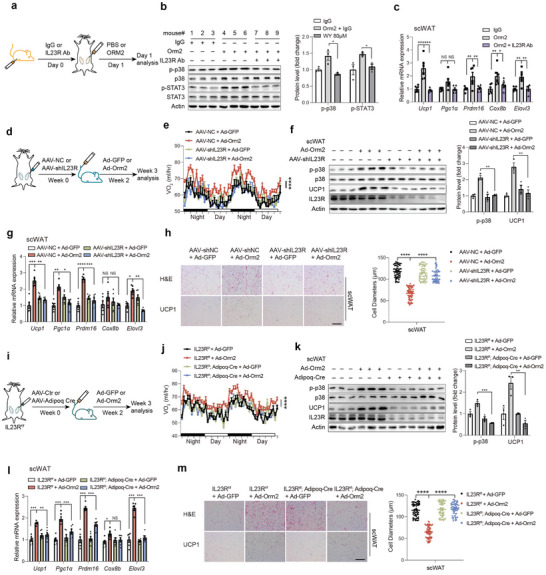
Orm2 promotes adipose browning through IL23R. a–c) C57BL/6 mice were subcutaneously injected with IL23R antibody or control IgG (500 µg kg^−1^), followed by an injection of recombinant Orm2 (5 mg kg^−1^) directly into the subcutaneous white adipose tissue (scWAT). Schematic depicting the workflow for the mice treatment (a) and representative immunoblots of STAT3 phosphorylation and p38 phosphorylation levels in scWAT (b), thermogenic genes mRNA levels in scWAT (c; n = 3) were detected. d–h) C57BL/6 mice were locally injected with AAV‐NC or AAV‐shIL23R in scWAT followed by injection of Ad‐GFP or Ad‐Orm2 intravenously. Schematic depicting the workflow for the mice treatment (d) and Oxygen consumption (VO_2_) (e; n = 6), immunoblots of p38 phosphorylation and Ucp1 protein levels in scWAT (f), thermogenic genes mRNA levels of scWAT (g; n = 6), H&E and Ucp1 staining of scWAT (h; Scale bar,100 µm, n = 3) were detected. i–m) IL23R ^f/f^ mice were locally injected with AAV‐Ctr or AAV‐Adipoq Cre into scWAT followed by injection of Ad‐GFP or Ad‐Orm2 intravenously. Schematic depicting the workflow for the mice treatment (i) and Oxygen consumption (VO_2_) (j; n = 6), immunoblots of p38 phosphorylation and Ucp1 protein levels in scWAT (k), thermogenic genes mRNA levels of scWAT (l; n = 6), H&E and Ucp1 staining of scWAT (m; Scale bar,100 µm) were detected. Significance was calculated by unpaired two‐tailed Student's t test (b, c, f‐h, k‐m) or two‐sided ANCOVA (e, j); *p < 0.05, **p < 0.01, ***p < 0.001 and ****p < 0.0001 were considered to be significant. Error bars represent the mean ± SEM.

To rigorously ascertain the contributory role of IL23R in mediating Orm2's biological effects in adipose tissue, IL23R‐neutralizing antibodies were administered subcutaneously, while recombinant Orm2 was directly injected into the scWAT of mice (**Figure** **4**a). Injection of Orm2 into the scWAT stimulated phosphorylation of p38 and STAT3, along with an increase in thermogenic gene expression, which were inhibited by co‐treatment with an IL23R‐specific antibody (Figure 4b,c). Similarly, hepatic overexpression of Orm2 via an adenoviral vector (Ad‐Orm2) led to enhanced browning of scWAT, as demonstrated by increased VO_2_, energy expenditure (EE), upregulation of thermogenic genes, and downregulation of respiratory exchange ratio (RER) (Figure 4d‐h; Figure S5a,b, Supporting Information). This browning effect was mitigated upon IL23R knockdown in scWAT, achieved by injection of an Adeno‐associated virus (AAV) vector expressing IL23R shRNA (Figure 4d‐h; Figure S5a, Supporting Information). Meanwhile, adipose tissue mass, food intake and activity levels were not influenced (Figure S5c‐e, Supporting Information).

We noted an increased IL23R expression during adipocyte differentiation (Figure S5f,g, Supporting Information). Besides, IL23R is predominantly expressed in CD4^+^ T cells, with much lower expression observed in B cells, NK cells, innate lymphoid cells, and myeloid cell subsets, which are also present in adipose tissue. To unequivocally demonstrate that Orm2‐induced adipose thermogenesis is mediated by IL23R expressed on adipocytes, adipocyte‐specific IL23R knockout (IL23R AKO) mice were created using AAV‐mediated delivery of Adipoq‐Cre to the scWAT of IL23R^flox/flox^ mice (Adipoq‐Cre x IL23R^f/f^). In line with previous findings, hepatic overexpression of Orm2 via Ad‐Orm2 injections induced browning in the adipose tissue of WT mice but failed to do so in IL23R AKO mice (Figure 4i‐m; Figure S5h,i, Supporting Information). Adipose tissue mass, food intake and activity levels remained unchanged across the groups. (Figure S5j‐l, Supporting Information). The findings provide compelling evidence that Orm2 enhances thermogenic processes specifically via IL23R‐GP130 signalling in adipocytes.

### 2.5 Orm2 Treatment Ameliorates HFD‐Induced Obesity

**Figure 5:**
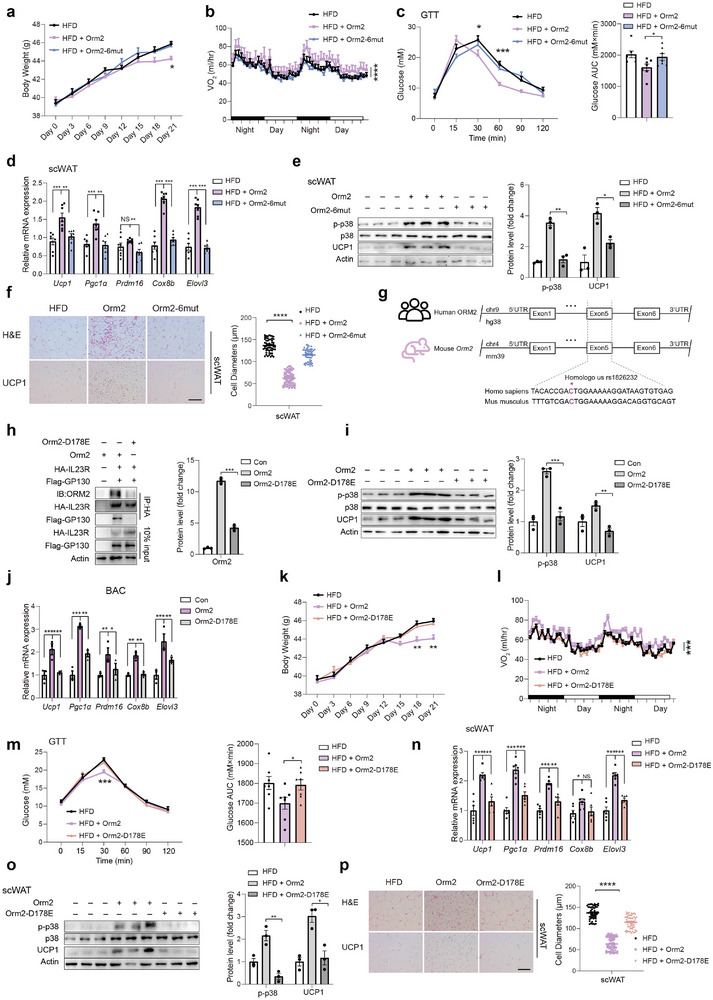
Orm2 treatment ameliorates HFD‐induced obesity. a–f) HFD‐fed mice were intraperitoneally injected with recombinant Orm2 protein, Orm2‐6mut protein 1.5 mg kg^−1^, or vehicle control (PBS) for 21 d. Body weight (a; n = 7), Oxygen consumption (VO_2_) (b; n = 7), Glucose tolerance test (c; n = 7), thermogenic genes mRNA levels of scWAT (d; n = 7), immunoblots of p38 phosphorylation and Ucp1 protein levels in scWAT (e), H&E and Ucp1 staining of scWAT (f; Scale bar,100 µm) were detected. g) Conserved motif module surrounding the rs1826232 locus in human and mouse species. UTR, untranslated region. h) Co‐immunoprecipitation analysis showing the interaction between Orm2 or Orm2‐D178E with transfected HA‐IL23R and Flag‐GP130 in HEK 293T cells. i,j) Representative immunoblots of p38 phosphorylation and Ucp1 protein levels (i) and thermogenic genes mRNA levels (j) in BAC incubated with Orm2 or Orm2‐D178E. k–p) HFD‐fed mice were intraperitoneally injected with recombinant Orm2 protein, Orm2‐D178E protein (1.5 mg kg^−1^), or vehicle control (PBS) for 21 d. Body weight (k; n = 7), Oxygen consumption (VO_2_) (l; n = 7), Glucose tolerance test (m; n = 7), thermogenic genes mRNA levels of scWAT (n; n = 7), immunoblots of p38 phosphorylation and Ucp1 protein levels in scWAT (o), H&E and Ucp1 staining of scWAT (p; Scale bar,100 µm) were detected. Significance was calculated by unpaired two‐tailed Student's t test (d‐f, h‐j, n‐p), two‐way ANOVA (a, c, k, m) or two‐sided ANCOVA (b, l); *p < 0.05, **p < 0.01, ***p < 0.001 and ****p < 0.0001 were considered to be significant. Error bars represent the mean ± SEM.

IF has been documented to mitigate obesity, which is at least partially attributed to the enhancement of adipose tissue browning and thermogenesis.^[^ ^11^, ^12^, ^13^ ^]^ Given the observed promotion of adipose browning by IF‐induced Orm2, we proceeded to evaluate the potential of Orm2 treatment in countering obesity. Mice subjected to a high‐fat diet (HFD) and weighing ≈40 g were administered local injections of either Orm2 protein or its mutant variant, Orm2‐6mut protein (1.5 mg kg^−1^ per day), directly into adipose tissue for a period of 21 days. It was found that Orm2, but not the Orm2‐6mut, significantly counteracted the body weight gain and led to a reduction in adipose tissue mass induced by the HFD (**Figure** **5**a; Figure S6a, Supporting Information). Notably, there were no observable changes in food intake or physical activity levels; however, an increase in oxygen consumption (VO2) and energy expenditure (EE), along with a decrease in the respiratory exchange ratio (RER), specifically recorded in the Orm2‐treated HFD mice, effects not seen with the Orm2‐6mut treatment. (Figure 5b; Figure S6b‐e, Supporting Information). Furthermore, the Orm2‐treated HFD mice displayed enhanced glucose tolerance. (Figure 5c). In addition, a marked upregulation in thermogenic gene expression and Ucp1 protein levels was elicited by Orm2, but not Orm2‐6mut in the scWAT (Figure 5d‐f).

We next explored the association of Orm2 genetic variants (single‐nucleotide polymorphisms, SNPs) with metabolic traits in humans. Using previously reported genome‐wide association study (GWAS) datasets, we identified two missense variants in exon of the Orm2 gene (Figure S6f, Supporting Information). By sequence alignment, we could identify a missense variant (C > A/T, rs1826232) in exon5 of the Orm2 gene is of great conservative (Figure 5g; Figure S6g, Supporting Information). Notably, the Orm2‐D178E mutant exhibited a reduced binding affinity to the GP130/IL23R complex (Figure 5h) and demonstrated diminished p38 phosphorylation as well as a compromised ability to stimulate thermogenic gene expression (Figure 5i,j). Upon administration to HFD‐fed mice, wild‐type Orm2, but not the Orm2‐D178E variant, was able to promote thermogenesis and mitigate the effects of obesity (Figure 5k‐p; Figure S6h‐l, Supporting Information). These results indicated the potential contribution of Orm2 mutation in human obesity.

While these results indicate a potential functional impact of the Orm2‐D178E variant, it is important to note that the current GWAS data do not show a statistically significant association between this SNP and obesity. Therefore, further research with larger cohorts and more detailed phenotypic analyses is necessary to fully understand the role of Orm2 genetic variants in human metabolic traits and obesity.

## 3 Discussion

Intermittent fasting (IF) has been widely recognized for its potent ability to enhance metabolic equilibrium, chiefly by attenuating inflammation and augmenting insulin sensitivity. The liver, a central metabolic organ, exhibits a pronounced response to IF. It has been reported that 12–24 h of fasting results in depletion of hepatic glycogen after that fat‐derived ketone bodies and free fatty acids are motivated as energy sources, suggesting that liver‐adipose communication is critical to achieve the health benefits of IF. Nonetheless, the specific underlying mechanisms facilitating this liver‐adipose tissue crosstalk have largely remained elusive. In this study, we have identified a mechanistic pathway facilitating crosstalk between liver and adipose tissue that serves to enhance thermogenesis during IF. Hepatic PPARA*α*‐mediated Orm2 expression in liver promoted adipose thermogenesis during IF. This effect was orchestrated through IL23R/GP130‐p38 cascade. Notably, recombinant Orm2 but not the IL23R/GP130‐binding mutant form ameliorated HFD‐induced obesity. For clinical significance, we found the missense variant rs1826232 has been identified in 38 GWAS results related to obesity, suggesting it might be essential for human. Although the association between rs1826232 with obesity is not significant enough to reach p value<0.05, we demonstrated that Orm2‐D178E compromised its function to ameliorate high‐fat‐diet‐induced obesity. These findings establish Orm2 as an IF‐induced hepatokine that plays a critical role in mediating the inter‐organ communication between the liver and adipose tissue.

Previous studies have indicated that Orm2 can be secreted into the bloodstream, yet its role as a circulating factor remains unclear. Orm2 is a significant glycoprotein predominantly synthesized and secreted by hepatocytes. In humans, ORM2 was significantly overexpressed in liver tissue, over 2000 times that of other tissues.^[^ ^38^ ^]^ Despite the predominant expression of Orm2 in liver tissue, there have been reports indicating that Orm2 can also be detected in non‐hepatic tissues, including astrocytes within the brain.^[^ ^39^ ^]^ Our findings corroborate that Orm2 is primarily expressed in the liver, with a significantly lower level of expression observed in adipose tissue. We have further substantiated that serum levels of Orm2 were elevated in mice subjected to IF as opposed to those on AL feeding. This increase correlated with an upsurge in Orm2 expression in the liver, but not in BAT or scWAT. Subsequently, we verified that Orm2 was capable of being secreted into culture medium by primary hepatocytes. These results suggested that liver is the major source of circulating Orm2 in IF‐treated mice, although we could not completely rule out the possibility that other tissue may also contribute to the circulating Orm2.

Orm2 and Orm1 exhibit a high degree of similarity, with an 89.6% sequence identity, and both function as acute‐phase proteins in the inflammatory response. Previous studies have demonstrated that Orm1 could bind directly to leptin receptor (LepR) and activate the receptor‐mediated JAK2‐STAT3 signalling in the hypothalamus tissue.^[^ ^17^ ^]^ In addition, Orm1 could also bind to C‐C chemokine receptor type 5 (CCR5) on muscle cells and activate Toll‐like receptor‐4 (TLR4) downstream signalling in immune cells respectively.^[^ ^28^, ^29^ ^]^ However, our data indicated that Orm2 promoted adipose thermogenesis independent of LepR, CCR5 and TLR4. Our research has identified IL23R/GP130 as a novel receptor for Orm2. IL23R/GP130, known as a component of the receptor complex for IL‐6 family cytokines such as IL‐6, CNTF, CT‐1, and OSM, plays a role in inflammatory regulation across various tissues. This discovery implicates IL23R/GP130 in the Orm2 signalling pathway, suggesting a previously unrecognized function in mediating adipose tissue thermogenesis. This could serve as a new mechanism to explain the inflammatory function of Orm2 and whether Orm1 could also function through IL23R/GP130 will need further investigation.

Interestingly, we confirmed that IL23R expression was markedly induced during adipocyte differentiation. At the meantime, knockout of IL23R in adipocytes abolished Orm2‐mediated increases in thermogenic gene expression, suggesting that adipocyte‐expressed IL23R at least partially mediated Orm2 function on thermogenesis. However, adipose tissues comprise various IL23R‐expressed immune cells such as CD4^+^ T cells, macrophages and myeloid cells, whether Orm2 might function through these cells to achieve the pro‐thermogenic effect will need further investigation.

Collectively, our research has uncovered a novel inter‐organ communication mechanism between liver and adipose during IF. Liver‐secreted Orm2 promoted thermogenesis in BAT and scWAT via p38 through binding to cell surface GP130/IL23R and administration of Orm2 ameliorated HFD‐induced obesity in vivo. Future clinical studies are needed to explore the therapeutic potential of Orm2 for the treatment of obesity in humans.

## 4 Experimental Section

### Animal Experiments

All mouse experiment were approved by the Animal Experiment Committee of Tongji University and were conducted in accordance with the guidelines of the School of Medicine. All animals were bred at room temperature (RT, 25 °C) and humidity‐controlled room with 12 h light/dark cycle. Standard normal diet (5% fat; Research Diet, D12450, New Brunswick, NJ, USA) and sterile water were given ad libitum. C57BL/6J mice were purchased from Slack Laboratory Animal Co., Ltd (SLAC, Shanghai, China). Whole‐body Orm2 knockout (KO) mice were kindly provided by Prof. Yan Lu. The IL23R^flox/flox^ mice were purchased from Cyagen Biosciences for the purpose of specific gene knockout studies.

### Cell Culture

Primary adipocytes differentiated from SVF of scWAT that was isolated from 6‐week‐old C57BL/6J male mice. Post confluent cells were induced to differentiate in DMEM/F12 (Yuanpei) containing 10% fetal bovine serum (FBS), 0.5 mm 3‐isobutyl‐1‐methylxanthine (IBMX), 1 µm dexamethasone, 10 µg mL^−1^ insulin, 1 nm T3, 125 nm indomethacin and 0.5 µm rosiglitazone for two days and then the cells were cultured in DMEM (10% FBS, 10 µg mL^−1^ insulin, 1 nm T3 and 1 µm rosiglitazone) for an additional two days. After these procedures, the cells were maintained in DMEM containing 10% FBS. Immortalized brown and beige lines (BAC) were obtained by infection with the retrovirus encoding SV40T antigen, kindly provided by Prof. Junli Liu (Shanghai Diabetes Institute, Department of Endocrinology and Metabolism, Shanghai Jiao Tong University Affiliated Sixth People's Hospital). The differentiation of BACs was the same as that of SVFs. HEK293A, HEK293T, and Hepa1‐6 cell lines were obtained from the American Type Culture Collection (ATCC) and cultured in DMEM containing 10% FBS and penicillin/streptomycin (PS) (100 U mL^−1^ and 100 µg mL^−1^). Primary hepatocytes were isolated from male mice by portal intravenous infusion of collagenase buffer. After removing the supernatant, cells were resuspended in M199 containing PS with 10% FBS and then seeded in a six‐well plate at a final density 0.5 × 10^6^ cells per well. All cell lines were routinely tested and found to be negative for mycoplasma contamination.

### Seahorse XF Mito Stress Test

Stromal vascular fraction (SVF) cells were induced to differentiate into mature beige adipocytes. Following differentiation, cells were seeded onto microplates provided in the Seahorse XF Cell Mito Stress Test Kit (Agilent Technologies, Santa Clara, CA) at an appropriate density to ensure confluent growth. After seeding, the mature beige adipocytes were treated with Orm2 at a concentration of 100 ng mL^−1^ for 24 h to stimulate specific cellular responses prior to analysis. On the day of the assay, the treated cells were washed and incubated in XF Base Medium, supplemented as per the kit's instructions with glucose, pyruvate, and L‐glutamine, at 37 °C in a non‐CO2 environment. The Seahorse XF Analyzer was used to perform the Cell Mito Stress Test which includes sequential injections of mitochondrial inhibitors: oligomycin (1.5 µm), FCCP (2 µm), and a combination of rotenone and antimycin A (0.5 µm). These compounds perturb mitochondrial function, facilitating the measurement of key parameters such as basal respiration, ATP production, and maximal respiratory capacity.

### mtDNA Measurement

Real‐time quantitative PCR (qPCR) was employed to measure the relative mitochondrial DNA (mtDNA) content. The beta‐2 microglobulin gene (B2M) was chosen as the genomic DNA (gDNA) normalizer to compute the mtDNA/gDNA ratio. A 322 bp region of mouse mtDNA was amplified using the forward primer mtDNAF (5′‐CGACCTCGATGTTGGATCA‐3′) and the reverse primer mtDNAR (5′‐AGAGGATTTGAACCTCTGG‐3′). For the B2M gene, a 106 bp fragment was amplified with the forward primer B2MF (5′‐TCTCTGCTCCCCACCTCTAAGT‐3′) and the reverse primer B2MR (5′‐TGCTGTCTCGATGTTTGATGTATCT‐3′). The data was presented as the ratio of the relative copy number of mitochondrial DNA (mtDNA) to genomic DNA (gDNA).

### Serum Biochemical Assays

The concentrations of serum non‐esterified fatty acids (NEFA) and glycerol were determined utilizing specific assay kits (A042‐2‐1 for NEFA and F005‐1‐1 for glycerol) supplied by Nanjing Jiancheng Bioengineering Institute, Nanjing, China. Additionally, ketone bodies were quantitatively assessed employing the Ketone Body Assay Kit BC5065, which was obtained from Solarbio Science & Technology, Beijing, China.

### IF Regimen

The study utilized a 2:1 intermittent fasting (IF) regimen, consisting of two days of ad libitum feeding alternated with a single day of fasting. This sequence was repeated for four complete cycles. During fasting periods, food was withdrawn at 12:00 AM and reinstated 24 h later, precisely at 12:00 AM the following day. All mice were fasted overnight before sacrifice at the end of the study.

### Adenovirus‐Mediated Overexpression in Mouse Liver

To overexpress the Orm2 protein in mouse liver, a recombinant adenoviral vector system acquired from Jikai Gene Technology Co., Ltd was utilized. For the purpose of adenoviral‐mediated gene delivery, 8‐week‐old male mice received a tail vein injection of 1×10^9^ plaque‐forming units (pfu) of either Ad‐Orm2 or Ad‐GFP. Seven days following the adenovirus injection, the mice were euthanized, and both tissue samples and plasma were harvested for subsequent analyses. The knockdown of PPAR*α* was achieved using an Ad‐shPPAR*α* following the same protocol previously described.

### AAV8‐Mediated Gene Expression and Knockdown

The AAV shRNA vector pAAV‐U6‐shNC‐CMV‐EGFP‐WPRE and AAV‐U6‐shIL23R‐EGFP‐WPRE (CGGAGGAATCACAAGTATAAA) were obtained from Obio Technology Co., Ltd. To examine the long‐term effects of IL23R disruption, C57BL/6J mice were administered AAV‐shIL23R or AAV‐shNC via local injection into scWAT and BAT depots, at a concentration of 1×10^11^ pfu. Following a two‐week recovery period, AAV‐treated mice were administered with either Ad‐Orm2 or Ad‐GFP for one week. All tests were performed 3–5 weeks after AAV injection.

### Recombinant Protein Treatment for HFD‐Induced Obesity in Mice

In this study, to investigate the effects of recombinant proteins on obesity induced by HFD, male C57BL/6 mice aged 8 weeks were subjected to HFD (60% fat, D12492, Research Diets) for 10 weeks and then treated with a 1.5 mg kg^−1^ recombinant protein via intraperitoneal injection. The dosage of the recombinant protein was calculated and adjusted based on the body weight of the mice, with injections administered daily for 21 days.

### Plasmid Injection

For plasmid injection, mice were weighed to determine the volume of delivery solution. Total volume mL = 10%$\frac{\mathrm{mouse}\;\mathrm{weight}\;(g)}{10\;g\;\mathrm{mL}-1}$+0.1 mL delivery solution.^[^ ^40^ ^]^ Mice were injected through tail vein injection within 4–8 s at a constant rate and euthanized at day 4.

### Reconstruction of Plasmids and Preparation of Lentivirus

The vector pLK0.1‐CMV‐copGFP‐PURO was used as a backbone plasmid to reconstruct lentiviral vector containing mouse GP130 shRNA#3 GCGTCTTGTTCTGCTTTAACA (Tsingke Biotechnology Co.,Ltd). The reconstructed plasmid was verified by Sanger sequencing. Lentiviral particles were produced by co‐transfecting HEK293T cells with shRNA‐bearing lentiviral vectors alongside packaging plasmids psPAX2 and pMD2.G. Culture supernatants collected at 48 and 72 h post‐transfection were filtered and concentrated with PEG 8000, followed by centrifugation at 4000 × *g* for 20 min at 4 °C.

### Conditioned Medium Preparation and Antibody Neutralization Experiment

To harvest the conditioned medium from treated hepatocytes, either primary hepatocytes or Hepa1‐6 cells were pre‐incubated with viral vectors or pharmacological agonists 24 h prior to the collection. Post‐treatment, the medium containing the agonist was removed, and the hepatocytes were carefully washed with phosphate‐buffered saline (PBS). The culture was then transitioned to a serum‐free medium to avoid interference from serum components in subsequent analyses of secreted factors. After a further 24‐h incubation period in serum‐free conditions, the supernatant was collected. The conditioned medium (CM) was then centrifuged at 15000 × *g* for 5 min to pellet any cellular residue and filtered through a 0.22 µm pore size filter to ensure the removal of any remaining particulate matter.

To neutralize specific proteins within the CM, relevant antibodies were added at a dilution of 1:1000. The mixture was incubated for 24 h at 4 °C to allow for adequate antibody‐protein interactions and neutralization. Following this incubation period, BAC cells were stimulated with the treated medium to assess the functional impact of antibody‐mediated neutralization.

### Luciferase Reporter Assay

HEK293T cells were transfected with Orm2‐Luc reporter, RSV‐*β* gal, and the indicated plasmids (pcDNA3.1‐HA‐PPAR*α*) for 48 h. In brief, cell lysates were collected and divided into two parts, one for luciferase assay, another for *β*‐galactosidase assay.

### Preparation of Orm2/Orm2‐6mut/Orm2‐D178E Protein from HEK293T Cells

HEK293T cells were transfected with pDC315‐Orm2‐HA using standard methods. Six hours after transfection, the media were removed and cells were washed twice in PBS, followed by incubation in DMEM containing 10% FBS and 1% PS for 48–72 h. Cells were collected and lysed using 1.5 mL lysis buffer (Tris‐HCl 50 mm, pH = 7.4, NaCl 150 mm, sodium deoxycholate 0.25%, NP‐40 1%, EDTA 1 mm, PMSF 1 mm, Aprotinin 1 mg mL^−1^, Leupeptin 1 mg mL^−1^, Pepstain 1 mg mL^−1^) on ice for 10 min and centrifuged at 10 000 g for 15 min. The protein Orm2 was purified with HA beads, filtered with a 0.22 µm filter (Milipore) and stored at −20 °C. For the production of the Orm2‐6mut and Orm2‐D178E proteins, HEK293T cells were transfected with distinct plasmids: pDC315‐Orm2‐6mut‐HA and pDC315‐Orm2‐HA‐6His, respectively. Each plasmid was designed to express the HA‐tagged Orm2‐6mut protein or the Orm2‐D178E protein with a 6xHis tag, facilitating subsequent detection and purification steps.

### Glucose Tolerance Test and Insulin Tolerance Test

For the glucose tolerance test (GTT), mice were fasted for 16 h and then received an intraperitoneal injection of glucose (1 g kg^−1^ body weight). For the insulin tolerance test (ITT), mice were fasted for 6 h before receiving an intraperitoneal injection of insulin (1 IU kg^−1^ body weight). Blood glucose levels were measured at 0, 15, 30, 60, 90, and 120 min after injection using a glucometer.

### RNA Preparation and Real‐Time PCR

Total RNA was extracted from tissue or cells using TRIzol reagent (Invitrogen, Carlsbad, CA, USA) and reverse transcribed using the FastQuant RT kit (Tiangen, KR106). Real‐time PCR was performed with the Supereal SYBR Green kit (Tiangen, FP209) on a LightCycler 96 instrument (Roche, Penzberg, Germany).

### Western Blots

Total proteins were extracted from tissues or cells using RIPA buffer, and 30 µg of protein from each sample was separated by SDS‐PAGE and transferred to NC membranes (Catalog No. P‐N66485). The membranes were blocked with 5% milk powder in TBST for 1 h, followed by incubation with the primary antibody overnight at 4 °C. After three washes with TBST, the membranes were incubated with the secondary antibody for 1 h at RT. Protein bands were visualized using a ChemiDoc imaging system (Bio‐Rad Laboratories).

### H&E Staining and Immunohistochemistry

For hematoxylin and eosin (H&E) staining, tissues were fixed in 4% paraformaldehyde at RT and embedded in paraffin. Sections were then cut at a thickness of 5 µm and stained using the standard H&E protocol. For immunohistochemistry, after antigen retrieval, tissues were blocked with 5% bovine serum albumin (BSA) for 30 min at RT, followed by incubation with an anti‐Ucp1 antibody (1:250, Abcam, ab234430) diluted in 5% BSA for 2 h. After incubation with a horseradish peroxidase (HRP)‐conjugated secondary antibody specific for rabbit IgG, the samples were visualized under a microscope.

### Immunoprecipitation

HEK 293T cells were co‐transfected with various plasmids and subsequently lysed using a mixture of RIPA buffer and protease inhibitor. Following centrifugation, the supernatants were incubated with anti‐Flag and anti‐HA agarose beads at 4 °C overnight. The beads were then washed four to five times with ice‐cold RIPA buffer. Orm2 was added to the beads at a concentration of 0.1 µg mL^−1^ and incubated for 2 h. After three additional washes with RIPA buffer, the beads were subjected to SDS‐PAGE, and the proteins were analyzed by Western blot using the appropriate antibodies.

### Indirect Calorimetry

Food intake, oxygen consumption (VO_2_), and locomotor activity were measured in a subgroup of mice using a Comprehensive Lab Animal Monitoring System (CLAMS) (Columbus Instruments, Columbus, OH). Briefly, individual male mice with ad libitum access to food and water were acclimatized to metabolic cages for 48 h prior to a 72‐h period of continuous recording, with data collected every 15 min. The O_2_ content of the sample air from each cage was determined using an open‐circuit Oxymax system, which passed the air through sensors to measure oxygen levels. The respiratory exchange ratio (RER) was calculated using the formula: RER = VCO₂ (l/h) / VO₂ (l/h).

### Molecular Docking

The structures of Orm2, GP130, and IL23R were predicted using AlphaFold. Molecular docking studies were conducted by designating the extracellular domains of GP130 and IL23R as the receptors and Orm2 as the ligand. This approach was utilized to map the binding interface on the Orm2‐GP130/IL23R complex. The amino acid residues T71, G113, K136, E138, H166, and D170 of Orm2 were identified as crucial for the interaction with GP130/IL23R.

### Quantification and Statistical Analyses

All data were presented as mean ± standard error of the mean (SEM) unless otherwise specified. Statistical analyses were performed using Prism 8 (GraphPad Software) and SPSS. Statistical analyses were conducted using an unpaired (two‐tailed) Student's *t*‐test or two‐way ANOVA. The assessment of thermogenesis and indirect calorimetry was analyzed with a two‐sided analysis of covariance (ANCOVA), incorporating body weight as a covariate, using SPSS as previously reported. Statistically significant differences were denoted as *p < 0.05, **p < 0.01, ***p < 0.001, and ****p < 0.0001. The specific statistical methods used for each figure were detailed within the respective figure legends.

## Conflict of Interest

The authors declare no conflict of interest.

## Author Contributions

X.Z. and X.W. contributed equally to this work. B.L. conceived and directed the study. X.Z., X.W., J.W., D.Z., L.D., J.H., Z.‐N.Z. and B.L. designed and performed experiments and analyzed the data. X.Z., Z.‐N.Z. and B.L. wrote the manuscript with comments from all the authors. All authors provided input and reviewed the manuscript.

## Supporting information



Supporting Information

Supplemental Tables

## Data Availability

The data that support the findings of this study are available from the corresponding author upon reasonable request.
