# Supplementary material for: Intermittent Fasting‐Induced Orm2 Promotes Adipose Browning via the GP130/IL23R‐p38 Cascade
Source: Adv Sci (Weinh). 2024 Sep 9;11(42):2407789. doi: 10.1002/advs.202407789 (PMC11558143; doi:10.1002/advs.202407789)
Supplement: Supplementary file 1 — Supporting Information [file ADVS-11-2407789-s002.pdf]

ADVANCED SCIENCE
Open Access

Supporting Information

for *Adv. Sci.*, DOI 10.1002/advs.202407789

Intermittent Fasting-Induced Orm2 Promotes Adipose Browning via the GP130/IL23R-p38 Cascade

Xuejuan Zhu, Xinran Wang, Jingang Wang, Lei Du, Zhen-Ning Zhang, Donglei Zhou, Junfeng Han* and Bing Luan**

Supplementary materials

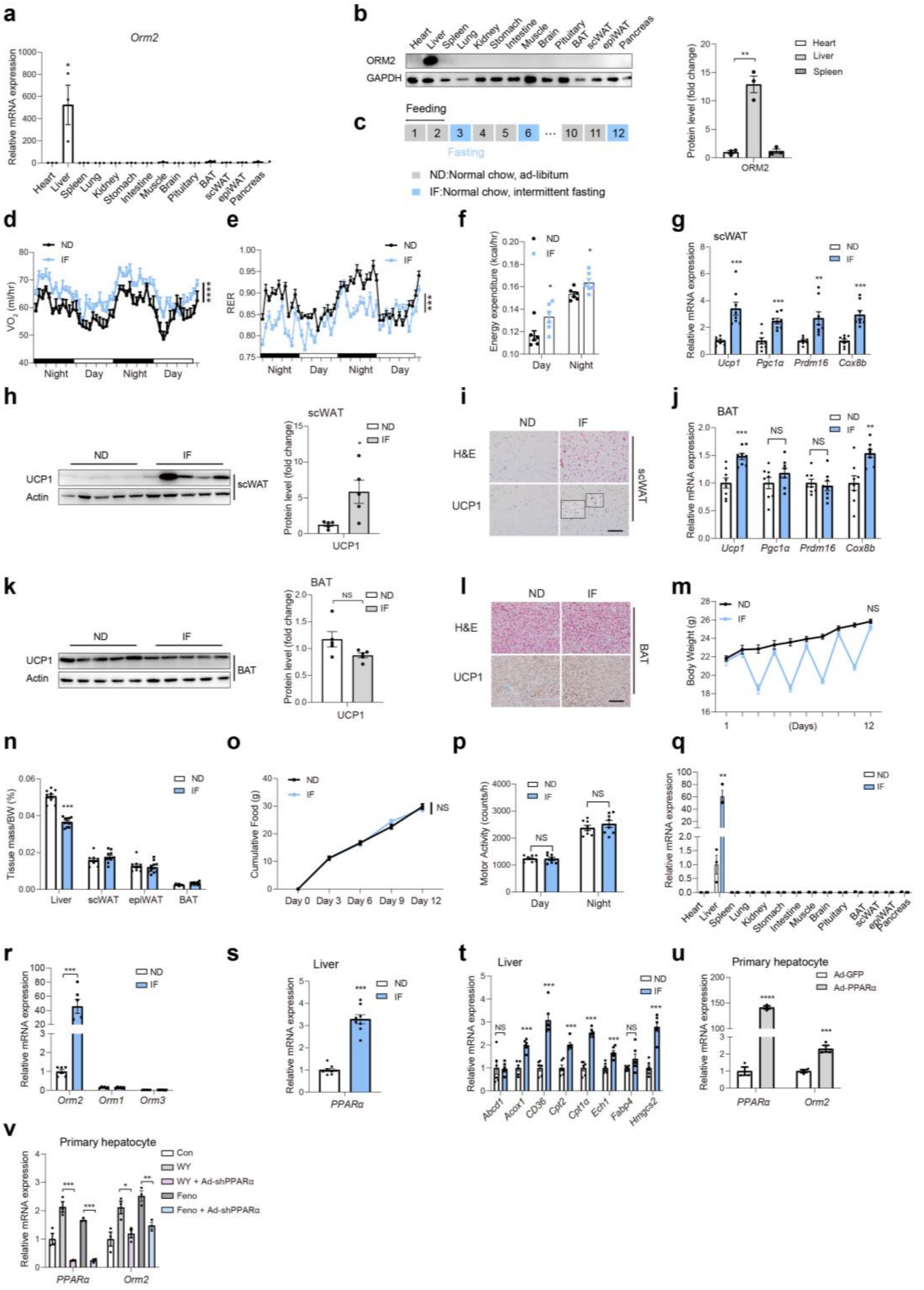

Figure S1. PPARα activation promotes hepatic Orm2 expression after IF

(a and b) Orm2 mRNA levels (a) and protein levels (b) in different tissues from wild-type (WT) mice (n = 3).

(c) Scheme illustrating the every-other-day intermittent fasting regimen.

(d-t) C57BL/6 mice were subjected to normal diet (ND) or Intermittent fasting (IF) for 12 days. Oxygen consumption (VO2) (d; n = 6), respiratory exchange ratio (RER) (e; n = 6); energy expenditure (EE) (f; n = 6); thermogenic gene expression of scWAT and BAT (g and j; n = 8), Ucp1 protein levels of scWAT and BAT (h and k), H&E and Ucp1 staining of scWAT and BAT (i and l; Scale bar,100 μm), body weight and tissue weight (m and n; n = 10), food intake (o; n = 7), activity (p; n = 8), Orm2 mRNA levels in different tissues (q; n = 6); Orm family gene expression (r; n = 6); PPARα mRNA levels in liver (s; n = 8); PPARα target genes mRNA levels in liver (t, n = 6) were detected.

(u) PPARα and Orm2 mRNA levels in primary hepatocytes infected with Ad-GFP or Ad-PPARα (n = 3).

(v) PPARα and Orm2 mRNA levels in primary hepatocytes treated with PPARα activator WY14643 (80 μM) (q) or Fenofibrate (60 μM) (r) after infection with Ad-shPPARα or Ad-shNC (n = 3).

Significance was calculated by unpaired two-tailed Student's t test (b, f-h, j, k, n, p-v), two-way ANOVA (e, m, o) or two-sided analysis of covariance (ANCOVA; d).; *p<0.05, **p<0.01, ***p<0.001 and ****p<0.0001 were considered to be significant. Error bars represent the mean ± SEM.

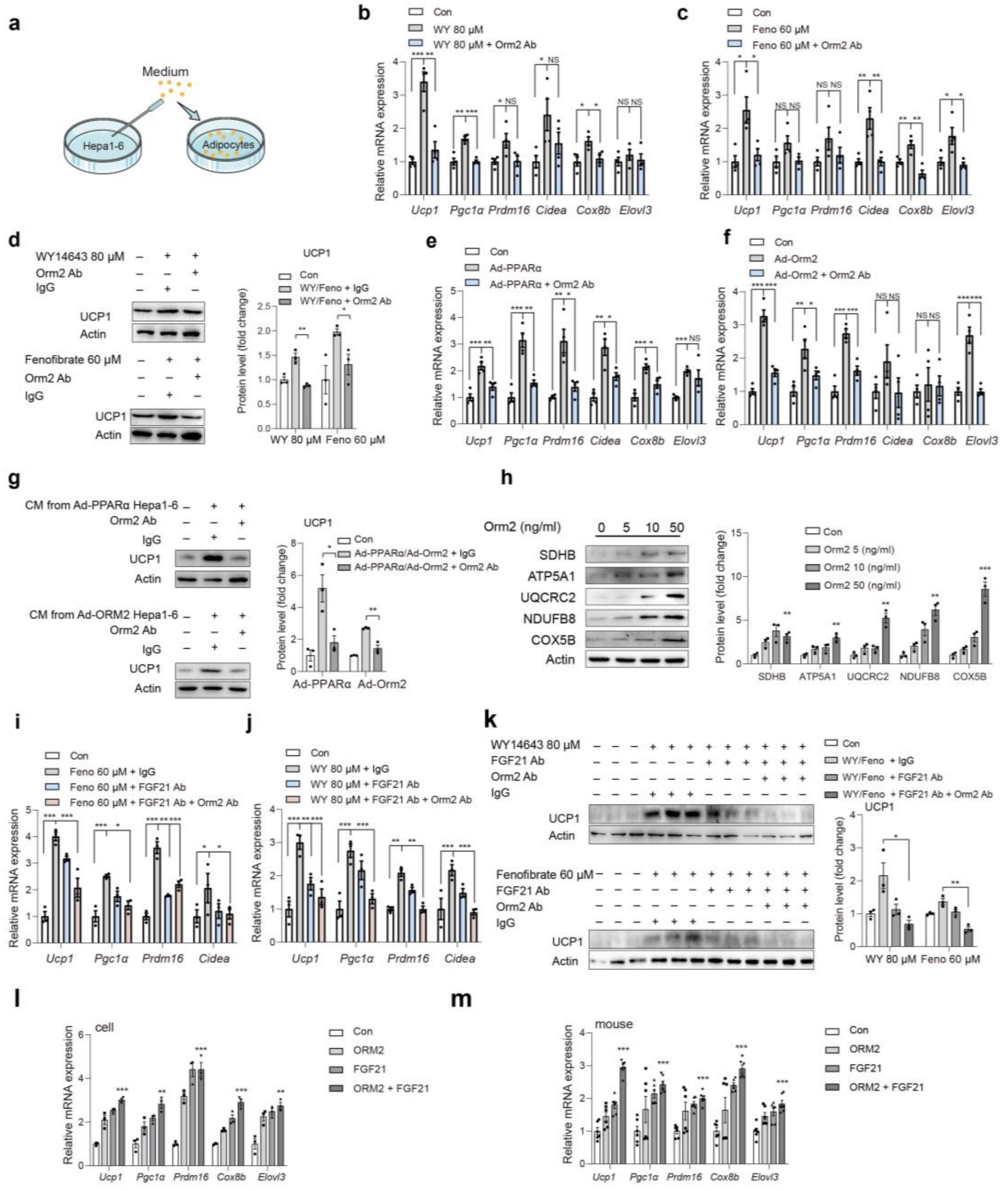

Figure S2 Orm2 enhance thermogenic and mitochondrial responses in adipocytes.

(a) Schematic depicting the workflow for the conditioned medium treatment. (b-d) Representative thermogenic genes mRNA levels (b and c; n = 3) and Ucp1 protein levels (d) in immortalized brown adipocytes (BAC) after exposure to conditioned medium (CM) collected from Hepa1-6 cells treated with WY14643 or Fenofibrate and Orm2 antibody for 12h.

(e-g) Representative thermogenic genes mRNA levels (e and f; n = 3) and Ucp1 protein levels (g) in BAC after exposure to conditioned mediums (CM) collected from Hepa1-6 cells treated with Ad-PPARα or Ad-Orm2 and Orm2 antibody for 12h.

(h) Representative immunoblots of several mitochondrial proteins (ATP5A1, SDHB, UQCR2, NDUFB8, COX5B) in adipocytes treated with Orm2 at concentrations of 0, 5, 10, and 50 ng/ml.

(i-k) Representative thermogenic genes mRNA levels (i and j; n = 3) and Ucp1 protein levels (k) in BAC after exposure to conditioned mediums (CM) collected from Hepa1-6 cells treated with Fenofibrate or WY14643 and FGF21 antibody or Orm2 antibody.

(l) Representative thermogenic genes mRNA levels in BAC treated with Orm2 (100 ng/ml) or FGF21 (100 ng/ml) for 12h (n = 3).

(m) Representative thermogenic genes mRNA levels in mouse scWAT after local injection of Orm2 (5 mg/kg) or FGF21 (5 mg/kg) for 24h (n = 6).

Significance was calculated by unpaired two-tailed Student's test (b-m), *p<0.05, **p<0.01 and ***p<0.001 were considered to be significant. Error bars represent the mean ± SEM.

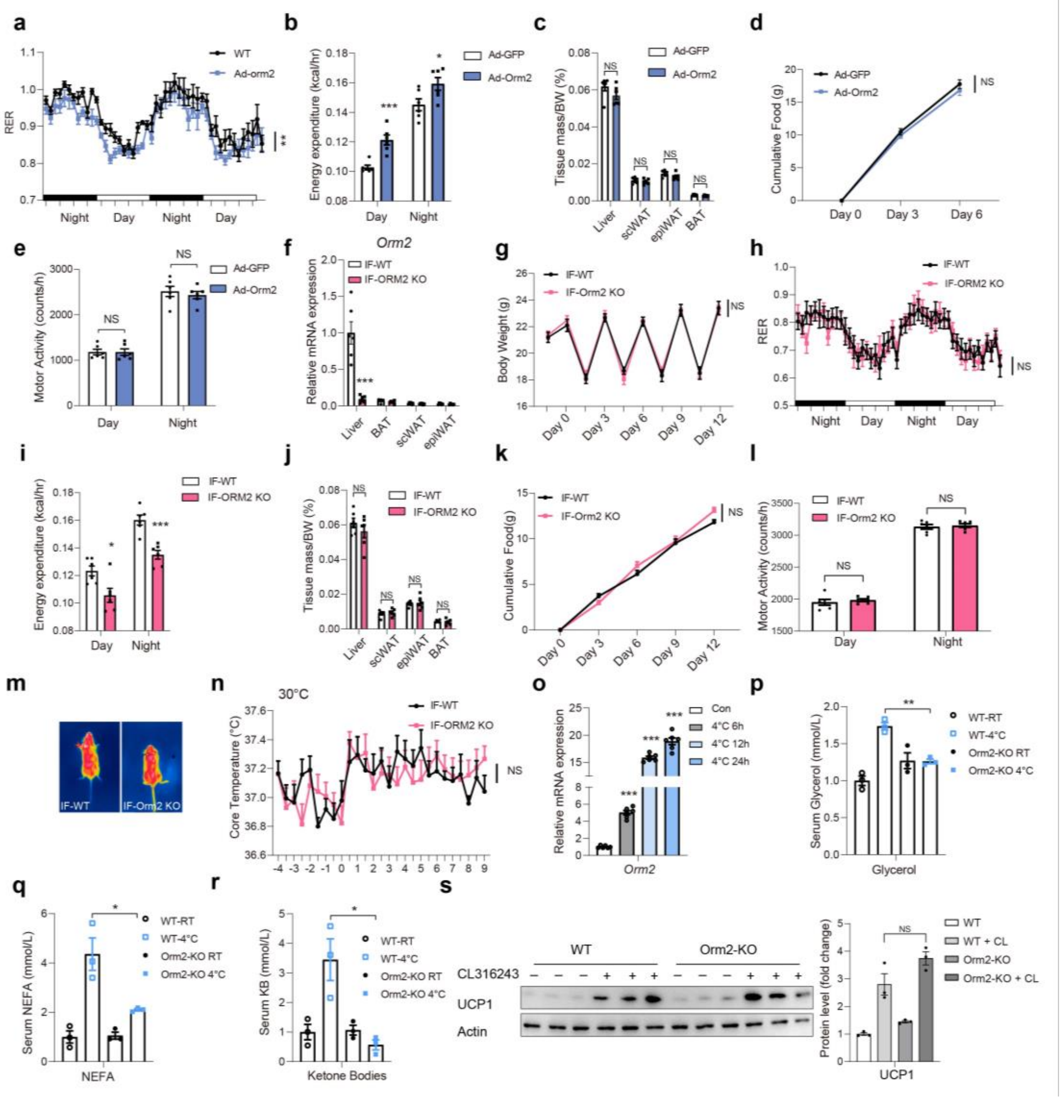

Figure S3 Orm2 promotes thermogenic program in scWAT

(a-e) Respiratory exchange ratio (RER) (a), energy expenditure (EE) (b), tissue mass (c), food intake (d) and activity (e) of Ad-GFP or Ad-Orm2 injected mice (n = 6).

(f) Orm2 mRNA levels in liver, bat, scWAT, and epiWAT of WT or Orm2-KO mice subjected to IF (n = 6).

(g-l) Body Weight (g), respiratory exchange ratio (RER) (h), energy expenditure (EE) (i), tissue mass (j), food intake (k) and activity (l) of WT or Orm2-KO mice subjected to IF (n = 6).

(m and n) Infrared thermography in WT or Orm2-KO mice that were subjected to IF for 12 days (m; n = 3); Core temperature after exposure to 30°C in WT and Orm2-KO mice (n; n = 6).

(o) Orm2 mRNA levels in mice that were subjected to 4°C 6h, 12 or 24h.

(p-r) Serum content of glycerol (p), NEFA (q), and ketone bodies (r) in WT and Orm2-KO mice treated at RT 4°C (n = 3).

(s) Ucp1 protein levels in primary beige adipocytes from WT or Orm2-KO mice after β3AR agonist CL316243 (1 μg/ml) treatment (n = 3).

Significance was calculated by unpaired two-tailed Student's test (b, c, e, f, i, j, l, o-s) or two-way ANOVA (a, d, g, h, k, n) *p<0.05, **p<0.01 and ***p<0.001 were considered to be significant. Error bars represent the mean ± SEM.

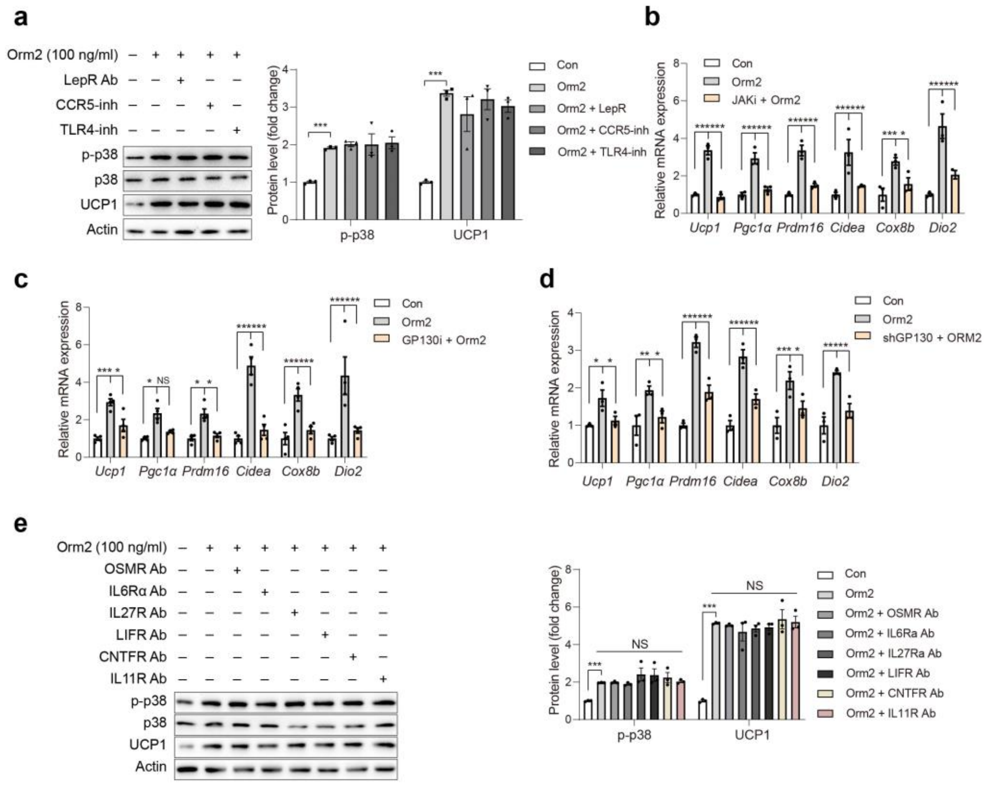

Figure S4 GP130-JAK-p38 cascade mediates Orm2 function

(a) Representative immunoblots of p38 phosphorylation and Ucp1 protein levels in BAC incubated with IgG, LepR antibody, CCR5 inhibitor Maraviroc (1 μM), or TLR4 inhibitor Resatorvid (1 μM) and treated with or without Orm2 (100 ng/ml).

(b-d) Representative thermogenic genes mRNA levels in BAC incubated with or without Orm2 (100 ng/ml), JAK inhibitor GLPG0634 (10 nM) (b; n = 3) and GP130 inhibitor SC144 (1 μM) (c; n = 3) or infected with Lentivirus-shGP130 (d; n = 3).

(e) Representative immunoblots of p38 phosphorylation and Ucp1 protein levels in BAC incubated with IgG, OSMR antibody, IL6R antibody, IL27R antibody, LIFR antibody, CNTFR antibody, or IL11R antibody with or without Orm2 (100 ng/ml) treatment.

Significance was calculated by unpaired two-tailed Student's t test (a-e); $^*p<0.05$, $^{**}p<0.01$ and $^{***}p<0.001$ were considered to be significant. Error bars represent the mean ± SEM.

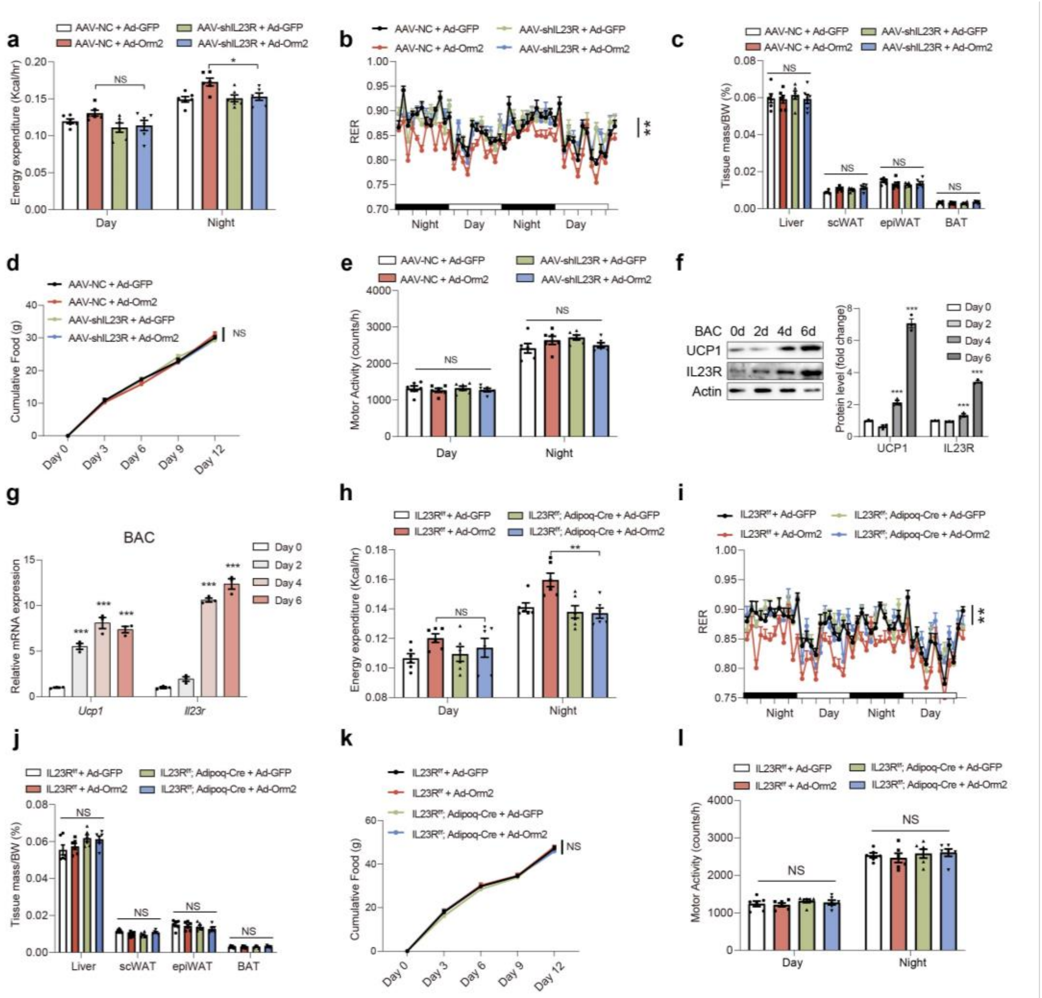

Figure S5 Orm2 functions through IL23R

(a-e) Energy expenditure (EE) (a), respiratory exchange ratio (RER) (b), tissue mass (c), food intake (d) and activity (e) of mouse injected with AAV-shIL23R or AAV-NC with or without Ad-GFP or Ad-Orm2 injection (n = 6).

(f and g) Ucp1 and IL23R protein levels (f) and mRNA levels (g; n = 3) during adipocyte differentiation.

(h-l) Energy expenditure (EE) (h), respiratory exchange ratio (RER) (i), tissue mass (j), food intake (k) and activity (l) of IL23R f/f mice injected with AAV-GFP or AAV-Adipoq Cre with or without Ad-GFP or Ad-Orm2 injection (n = 6).

Significance was calculated by unpaired two-tailed Student's t test (a, c, e-h, j, l), two-way ANOVA (b, d, i, k); $*p<0.05$, $**p<0.01$ and $***p<0.001$ were considered to be significant. Error bars represent the mean ± SEM.

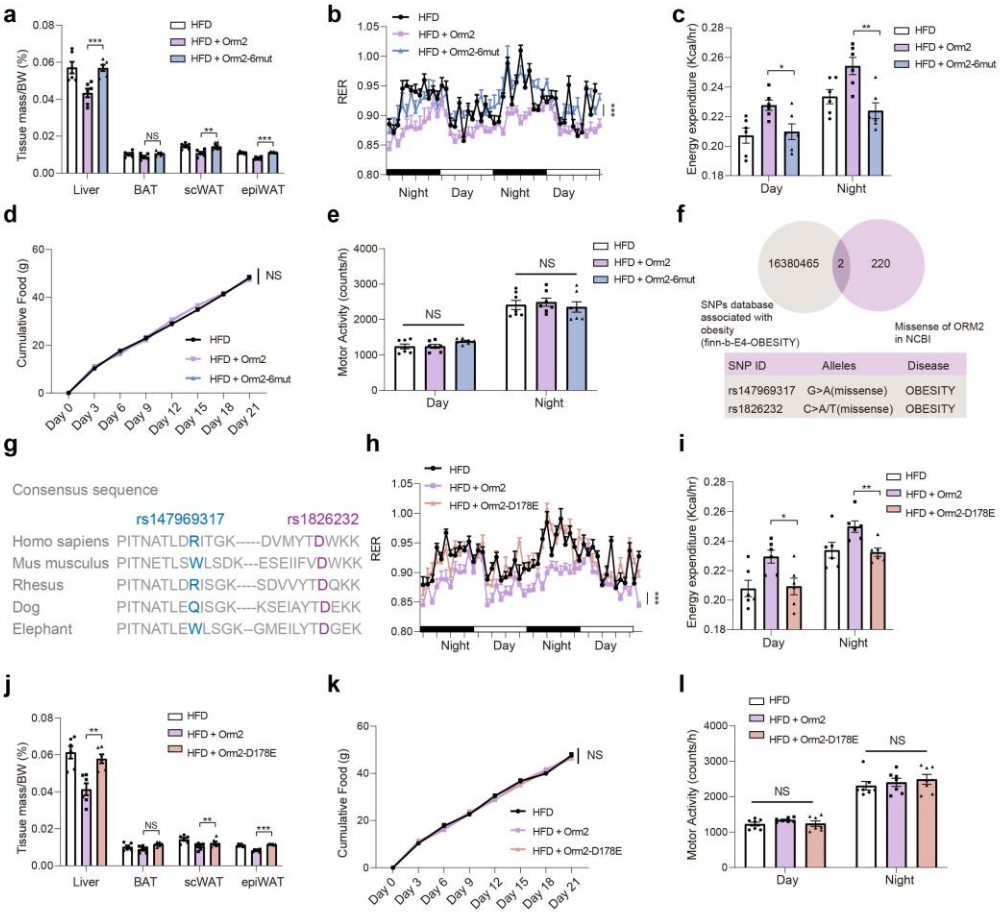

Figure S6. Orm2 does not influence food intake and physical activity in mice

(a-e) Tissue mass (a, n = 6), respiratory exchange ratio (RER) (b, n = 6), energy expenditure (EE) (c, n = 6), food intake (d, n = 7) and activity (e, n = 7) of HFD-fed mice injected with recombinant Orm2 protein, Orm2-6mut protein (1.5 mg/kg).

(f) Combine the obesity-related SNP dataset (finn-b-E4-OBESITY) in the Open GWAS database (https://gwas.mrcieu.ac.uk/) with the NCBI dbSNP database (https://www.ncbi.nlm.nih.gov/snp/?term=) for Orm2 SNV data for intersection analysis.

(g) Sequence conservation of rs147969317 (blue) and rs1826232 (purple).

(h-l) Respiratory exchange ratio (RER) (h, n = 6), energy expenditure (EE) (i, n = 6), tissue mass (j, n = 6), food intake (k, n = 7) and activity (l, n = 7) of HFD-fed mice injected injected with recombinant Orm2 protein, Orm2-D178E protein (1.5 mg/kg).

Significance was calculated by unpaired two-tailed Student's t test (a, c, e, i, j, l), or two-way ANOVA (b, d, h, k) *p<0.05, **p<0.01 and ***p<0.001 were considered to be significant. Error bars represent the mean ± SEM.
